# Addendum: Mehta et al. Standing Up for Learning: A Pilot Investigation on the Neurocognitive Benefits of Stand-Biased School Desks. *Int. J. Environ. Res. Public Health* 2016, *13*(1), 59; doi:10.3390/ijerph13010059

**DOI:** 10.3390/ijerph15030532

**Published:** 2018-03-16

**Authors:** Ranjana K. Mehta, Ashley E. Shortz, Mark E. Benden

**Affiliations:** Texas A&M School of Public Health, Environmental and Occupational Health, 1266 TAMU, College Station, TX 77843-1266, USA; ashortz@sph.tamhsc.edu (A.E.S.); mbenden@tamhsc.edu (M.E.B.)

The authors wish to update the Introduction in their paper published in the *International Journal of Environmental Research and Public Health (IJERPH)* [1]. 

In the middle of the third paragraph in the Introduction, they would like add a citation to the following sentence:

For example, the ability to mentally conceptualize a problem, store information temporarily in the visual-spatial sketchpad, develop a plan, evaluate and adapt complex goal-directed behavior has been identified as a product of working memory and executive function [2].

The changes do not affect the results. The manuscript will be updated and the original will remain online on the article webpage, with a reference to this addendum.
